# Autoantibody Signaling in Pemphigus Vulgaris: Development of an Integrated Model

**DOI:** 10.3389/fimmu.2018.00692

**Published:** 2018-04-19

**Authors:** Thomas Sajda, Animesh A. Sinha

**Affiliations:** Department of Dermatology, Jacobs School of Medicine and Biomedical Sciences, University at Buffalo, Buffalo, NY, United States

**Keywords:** pemphigus vulgaris, autoantibodies, signaling pathways, p38MAPK, calcium, epidermal growth factor receptor, Rho GTPases

## Abstract

Pemphigus vulgaris (PV) is an autoimmune skin blistering disease effecting both cutaneous and mucosal epithelia. Blister formation in PV is known to result from the binding of autoantibodies (autoAbs) to keratinocyte antigens. The primary antigenic targets of pathogenic autoAbs are known to be desmoglein 3, and to a lesser extent, desmoglein 1, cadherin family proteins that partially comprise the desmosome, a protein structure responsible for maintaining cell adhesion, although additional autoAbs, whose role in blister formation is still unclear, are also known to be present in PV patients. Nevertheless, there remain large gaps in knowledge concerning the precise mechanisms through which autoAb binding induces blister formation. Consequently, the primary therapeutic interventions for PV focus on systemic immunosuppression, whose side effects represent a significant health risk to patients. In an effort to identify novel, disease-specific therapeutic targets, a multitude of studies attempting to elucidate the pathogenic mechanisms downstream of autoAb binding, have led to significant advancements in the understanding of autoAb-mediated blister formation. Despite this enhanced characterization of disease processes, a satisfactory explanation of autoAb-induced acantholysis still does not exist. Here, we carefully review the literature investigating the pathogenic disease mechanisms in PV and, taking into account the full scope of results from these studies, provide a novel, comprehensive theory of blister formation in PV.

## Introduction

Pemphigus vulgaris (PV) is an autoimmune skin blistering disease characterized by the presence of autoantibodies (autoAbs) directed against keratinocyte surface antigens. It has been well established that autoAbs alone are capable of driving blister formation in PV. Early studies identified the primary target of pathogenic autoAbs as desmoglein 3 (Dsg3) and to a lesser extent, desmoglein 1 (Dsg1). More recently, additional autoAbs specificities have been identified in PV patients that could potentially also contribute to disease pathogenicity (1, 2). Despite extensive research, the exact mechanisms through which autoAbs induce a loss of cell–cell adhesion (also termed acantholysis) are not well understood.

Since the primary target of PV autoAbs was shown to be a desmosomal protein, most of the earliest theories of acantholysis suggested that the loss of cell adhesion was simply the result of autoAbs sterically hindering the homo- and heterophillic binding of desmosomal proteins between neighboring cells (3, 4). Studies showing that staphylococcal exfoliative toxins, which cleave Dsg1, could produce blisters similar to those seen in PF, indicated that disturbance of desmosomal proteins was capable of causing a loss of cell–cell adhesion (5, 6). The relationship between desmoglein expression and cell adhesion was further supported by the observation that Dsg3-deficient mice develop mucosal lesions similar to those seen in PV patients (7). Together, these data demonstrated that interference with desmoglein interactions was sufficient to drive a loss of cell–cell adhesion.

The observation by multiple groups that PVIgG was seen only to bind at desmosomal areas also supported the idea that autoAb interference of desmosomal interaction drives blister formation (8–10). In addition, in areas of acantholysis, PVIgG was seen to bind to both desmosomal plaques and split desmosomes (11), indicating that PVIgG could access desmosome-associated desmogleins.

Further support for the steric hindrance theory came from later studies attempting to characterize the fine-epitope specificity of anti-Dsg3 autoAbs. It was shown that autoAbs primarily target the amino terminal of Dsg3 which, based on the crystal structure of other classical cadherins, was predicted to facilitate trans-interaction (12–17). Furthermore, only anti-Dsg3 autoAbs targeting the amino terminal EC1-2 of Dsg3 could illicit blister formation when passively transferred to neonatal mice, not anti-Dsg3 autoAbs targeting the more carboxyl domains EC3-5. Another study demonstrated that anti-Dsg3 autoAbs preferentially recognize the mature form of Dsg3, but not an immature form, which requires additional proteolytic processing to participate in adhesion (18, 19). It was also shown that autoAb binding was dependent on the proper calcium stabilized formation of Dsg3, which is known to be required for proper adhesive functioning (13, 20, 21). Later experiments using atomic force microscopy (AFM) were able to demonstrate that PVIgG could directly interfere with Dsg3 trans-interaction (22).

One potential liability of the steric hindrance theory was the possibility that blistering effects were mediated by the constant regions of the Abs. However, experiments showing that Fab and F(ab_2_) fragments, as well as single-chain variable fragments which lack the Fc domain, were able to induce blister formation *in vitro* proved that Fc-dependent mechanisms were not necessary for blister formation (23–26). Additional experiments demonstrating the pathogenicity of PVIgG in C5a-deficient mice indicated that compliment activation was not required for acantholysis (23).

Over time, evidence has accumulated suggesting steric hindrance may not be the primary or sole pathogenic mechanism operative in PV. One of the earliest indications that alternative mechanisms may drive pathogenesis was the observation that IgG from PF patients could induce disease in mice without interfering with trans-adhesion of Dsg1 (27). It was noted in multiple studies that PVIgG was seen to bind extra-desmosomal spaces on the surface of keratinocytes, allowing for the possibility that binding of autoAbs outside of desmosomes may affect disease (3, 28). It was also shown that PVIgG binding induced cytoskeletal changes and the retraction of keratin intermediate filaments before any visible changes in desmosomes (29–33). It was also noted that in early PV lesions keratinocytes first separate at inter-desmosomal areas and desmosomes are still intact and interacting with neighboring desmosomes (29, 34–36). Together, these findings suggested that desmosomal separation may be downstream of other processes induced by the binding of autoAbs. Recently, one research group used AFM to demonstrate that the loss of Dsg3 binding alone was not sufficient to cause a loss of cell adhesion, strongly indicating that steric hindrance by itself cannot sufficiently explain acantholysis in PV (37).

An early alternative to the steric hindrance theory was suggested by results showing that the binding of autoAbs initiated the activation of proteases which in turn degraded Dsg3 and inhibited cell–cell adhesion. Specifically, plasminogen activator was thought to play a role in disease (38). PVIgG was shown to induce signaling that led to increased production of plasminogen activator (39, 40). Furthermore, PVIgG induced keratinocyte expression of plasminogen activator receptor (38, 41). However, inhibition of plasminogen *via* dexamethasone did not prevent PVIgG-induced acantholysis (42). The role of other proteases was also shown not to be essential in disease by the failure of protease inhibitors and gene ablation to prevent blister formation (43, 44).

One of the earliest studies that indicated that autoAbs may exert their pathogenic effect through the activation of intracellular cascades demonstrated that plakoglobin (Pkg)-deficient mice were protected from PVIgG-induced blister formation (45). Pkg, an armadillo family protein, is well established as a major signaling molecule involved in the regulation of cell adhesion (46, 47). The inability of PVIgG to induce blisters in the absence of Pkg strongly suggests that alteration of Pkg signaling is a primary pathogenic mechanism of PVIgG. In addition, keratinocytes incubated at 4°C did not show any effects of PVIgG on cell adhesion, suggesting that the mechanisms underlying blister formation are energy dependent (48).

Identification and characterization of the precise signaling pathways driving autoAb-induced acantholysis has been a significant focus for PV research. As a result, large amounts of (often conflicting) information concerning the signaling alterations downstream of anti-Dsg and PVIgG binding have been characterized. Moreover, studies showing that autoAbs in PV sera directed at non-desmoglein antigens can also elicit intracellular signaling have further complicated efforts to elicit the precise mechanisms driving disease (49, 50). The primary signaling pathways and the evidence that supports their role in PV pathogenesis are reviewed below (see Table 1 for evidence supporting steric hindrance vs. intracellular signaling).

**Table 1:** Evidence supporting steric hindrance vs. intracellular signaling.

Evidence supporting the steric hindrance theory
Desmoglein 3 (Dsg3)/desmoglein 1 (Dsg1), the primary targets of pathogenic autoantibodies (autoAbs), directly mediate cell adhesion Enzymatic cleavage of Dsg1 is sufficient to cause a loss of cell adhesion Pathogenic anti-Dsg3 autoAbs preferentially target the EC regions thought to mediate trans-adhesion Pathogenic anti-Dsg3 autoAbs preferentially recognize Dsg3 in the calcium bound, functional competent conformation Anti-Dsg3 autoAbs can access and bind Dsg3 molecules in intact desmosomes

**Evidence indicating a role for intracellular signaling**

In PF, a related disease, autoAbs targeting Dsg regions that do not mediate trans-adhesion can induce a loss of cell adhesion After exposure to PVIgG, cytoskeletal changes occur before impairment of desmosomal adhesion In early pemphigus vulgaris lesions, inter-desmosomal contacts are impaired while desmosomal contacts remain intact Studies using atomic force microscopy have shown that blocking of trans-adhesion alone does not induce a loss of cell adhesion PVIgG-induced acantholysis is impaired at low temperatures, suggesting an energy requiring process is involved Inhibition of multiple signaling pathways can inhibit PVIgG-induced acantholysis both *in vitro* and *in vivo*

## Signaling Pathways Implicated in PV

### p38MAPK

p38 is one of the three major families of mitogen-activated protein kinases (MAPK), which are known to play a prominent role in a wide range of cellular pathways (51). In general, p38MAPK proteins can be activated by environmental stress and regulate the transcription of inflammatory cytokines (52). All MAPKs require dual phosphorylation for enzymatic activity, and each contains a characteristic dual phosphorylation sequence which affect both the substrate specificity and ability to auto-phosphorylate (53, 54). There are four types of p38MAPK: α, β, γ, and δ, each displaying unique patterns of tissue expression (52). Only p38MAPK α, β, and δ are known to be expressed in keratinocytes (52, 54–58) and have been primarily associated with differentiation and apoptosis (59).

The significance of p38MAPK signaling in PV pathogenesis was first suggested by a study which observed that PVIgG induced significant increases in the phosphorylation of p38MAPK, MAPKAP2 (MK2), and heat-shock protein (Hsp)27 (60). The degree of phosphorylation was shown to increase when cells were treated with higher concentrations of PVIgG. In addition, treatment of cells with p38MAPK inhibitors was able to prevent PVIgG-induced acantholysis as well as changes in the actin cytoskeleton and the retraction of KIFs from desmosomal attachments, both of which are hallmarks of acantholysis in PV. Inhibition of p38MAPK also prevented PVIgG-induced phosphorylation of MK2, Hsp27, and p38MAPK (60).

Other studies assessing the role of PVIgG on p38MAPK activation identified that PVIgG causes keratin retraction and p38MAPK activation within 30 min, and another peak at 6–10 h in cultured human keratinocytes. Only inhibition at the earlier time point was associated with prevention of blister formation and keratin retraction (61). Another study showed that AK23, a mouse-derived monoclonal anti-Dsg3 antibody, could also activate p38MAPK, demonstrating that autoAb binding to Dsg3 specifically can lead to p38MAPK activation (62).

The relevance of p38MAPK in disease was emphasized by findings that both p38MAPK and Hsp27 are phosphorylated in the lesional skin of PV patients (63). Further studies demonstrated that p38MAPK inhibitor blocks acantholysis *in vivo*, as well as p38MAPK activation, suggesting auto-phosphorylation of p38MAPK (64). p38MAPK activation was also shown to cause Dsg3 internalization, and p38MAPK inhibition can prevent this phenomenon *in vivo* (65). A more detailed assessment of the effects of p38MAPK showed that p38 depletes extra-desmosomal Dsg3 early as 30 min, and also is responsible for later depletion (2–24 h) of other desmosomal cadherins as well as DP (66–70).

The regulation of cytoskeletal changes by p38MAPK is especially relevant in understanding how p38MAPK plays a role in PV pathogenesis. In epithelial cells, cell detachment has been shown to induce p38MAPK activation (71), indicating a close relationship between p38MAPK and cellular adhesion. Furthermore, p38MAPK activation can lead to phosphorylation, and subsequent destabilization of keratin intermediate filaments (72), which could be one explanation for the characteristic retraction of KIFs seen in PV. p38MAPK is known to regulate actin filaments as well (73). Since extra-desmosomal Dsg3 complexes with actin cytoskeleton and is required to bring DP to desmosomal plaques (74, 75), it is possible that PVIgG-induced dysregulation of p38MAPK could interfere with proper desmosome assembly.

MAPKAP2 is phosphorylated and activated by p38MAPK (76, 77). The activation of MK2 has been associated with cell cycle control, cytokine production, and regulation of the keratin and actin cytoskeletons (78–80). The inhibition of MK2 has been shown to prevent PVIgG-induced spontaneous blister formation in mice, but not blistering solicited *via* the application of mechanical stress (81). This suggests that while MK2 may mediate some of the pathogenic effects of PVIgG, additional pathways downstream of p38MAK are likely also contributing to acantholysis.

Heat-shock protein 27 is another signaling molecule activated by MK2 (82). Hsp27 regulates both actin (83–85) and keratin cytoskeleton (86, 87). Both p38MAPK and MK2 regulate the effect of Hsp27 on the cytoskeleton *via* phosphorylation (88–90). Taken together, these findings demonstrate that one possible pathogenic mechanism of PVIgG-induced p38MAPK activation could be the perversion of typical cytoskeletal regulation, resulting in impaired cell adhesion.

The degree of evidence supporting a role for p38MAPK activation in the pathogenesis of PV has led researchers to investigate the utility of p38MAPK inhibition for the clinical treatment of PV. In a small clinical trial, 15 PV patients were treated with KC-706, a small molecule allosteric inhibitor of p38MAPK. Unfortunately, the trial was terminated before completion due to severe side effects of the drug. At the time of cessation, half of the patients were seen to exhibit at least a partial response to treatment, whereas the other half showed no improvement or a worsening of symptoms (91). Hopefully, the development of newer, more specific inhibitors of p38MAPK and other downstream targets will allow for effective pathway inhibition while avoiding serious side effects (92).

In addition to the effects listed above, p38MAPK can affect epidermal growth factor receptor (EGFR) signaling (93), RhoA activation (60, 68, 94, 95), and various apoptotic pathways (96). All of these pathways have been implicated in PV pathogenesis and are discussed in greater detail below.

### Calcium/Protein Kinase C (PKC)/Phospholipase C (PLC)

The role of calcium signaling in keratinocyte differentiation and adhesion is well established. Increasing the Ca^2+^ concentration in keratinocyte culture medium increases intracellular calcium which in turn induces cell–cell contact (mainly adherens junctions) within 5 min, and formation of desmosomes within 2 h (97–104). PLC, an isoenzyme that is responsible for the cleavage of phosphatidylinositol 4,5-bisphosphate (PIP_2_) into inositol 1,4,5-triphosphate (IP_3_) and diacylglycerol (DAG), plays a significant role in calcium-induced keratinocyte differentiation (103, 105–108). PKC is a downstream target of PLC and is activated by calcium and DAG. Of the five isoforms known to be expressed in keratinocytes, PKC-alpha has been shown to play a major role in epidermal differentiation and proliferation (109–112).

Early studies showing that PVIgG leads to a rapid increase in intracellular calcium in keratinocytes were the first to implicate calcium signaling as a pathogenic signaling pathway in PV (113, 114). Additional studies demonstrated that PKC was activated within 30 s of treatment of PVIgG (115). A significant role for calcium signaling in PV pathogenesis was strengthened by studies which showed inhibition of PKC could prevent acantholysis both *in vitro* and *in vivo* (116, 117). It was also shown that inhibition of PLC prevented PVIgG-induced acantholysis, as well as increases in intracellular calcium and PKC activation (118). These results suggest that PVIgGs may exert their pathogenic effect by eliciting an increase in intracellular calcium, which leads to the activation of downstream signaling pathways.

The identification of PKC as a potential driver of PVIgG-induced pathogenesis is especially interesting due to the well-established role of PKC in cell adhesion. It has been shown that PKC activation leads to weakened cell–cell adhesion, whereas PKC inhibition results in increased adhesion (119, 120). In addition, PKC is known to be required for desmosome assembly and disassembly (41, 97, 121–126). The association of PKC with cell adhesion and desmosomal regulation may provide insight to help identify exactly how PVIgG induced PKC activation results in a loss of cell adhesion. One study showed that PVIgG-induced activation of PKC leads to its dissociation from KIFs and subsequent phosphorylation of DP, which resulted in desmosomal instability (127, 128). Another mechanism through which PKC activation may contribute to disease pathogenesis is by its effect on KIF turnover. It has been shown that PKC directly phosphorylates keratin molecules, leading to a turnover in KIFs (129, 130). This phenomenon may also explain the mechanistic background for the detachment of KIFs which is the hallmark of acantholysis.

### Epidermal Growth Factor Receptor

Epidermal growth factor receptor is a well-studied signaling pathway that impacts a multitude of cellular processes either through the direct binding of its ligand, epidermal growth factor, or by cross-activation from a number of other signaling pathways (131–134). EGFR signaling has been shown to impact cell adhesion *via* both adherens junctions and desmosomes (135–137). Specifically, association with Dsg1 has been shown to suppress EGFR extracellular signal-regulated kinase 1/2 signaling in skin (138). In addition, it has been shown that EGFR activation can induce the phosphorylation of Pkg and decrease the association of desmoplakin with the desmosome, resulting in weakened cellular adhesion (139). In general, these studies associate an activation of EGFR signaling with destabilization of desmosomal adhesion.

The association between EGFR signaling and cell adhesion provided a rationale for researchers to investigate if EGFR signaling played a role in acantholysis, and it was eventually shown that PVIgG led to the activation of EGFR in keratinocytes (49, 93). Further studies determined that the activation of EGFR could be detected as early as 30 min after exposure to PVIgG, but the activation occurred downstream of p38MAPK activation (49, 66, 67, 140). Another group was able to show that anti-Dsg3 autoAbs could also lead to EGFR activation (14). Multiple studies then showed that the inhibition of EGFR could prevent PVIgG-induced skin blistering both *in vivo* and in human skin explants (66, 93, 141).

In addition, EGFR is implicated in PV pathogenesis *via* a second signaling axis independent of p38MAPK. PVIgG binding induces secretion of EGF and related mediators from basal keratinocytes, which in turn activate Src, focal adhesion kinase, and mammalian target of rapamycin (mTOR) *via* EGFR and nitric oxide synthase. The end result is the activation of caspases 3 and 9, which have been proposed to contribute to bister formation (142–145). The role for traditional apoptosis in PV pathogenesis is currently unclear; our group showed that the induction of apoptosis-related mechanisms after anti-Dsg antibody binding is reversible and independent of the Fas/FasL axis (146). However, studies showing that inhibition of the low level caspase-3 induction caused by PVIgG prevented acantholysis *in vitro* and *in vivo* suggest that caspase-3 activation does indeed play a role in disease (147).

### Rho Family GTPases

Rho and Rac are both members of the Rho small GTPases family, which are known to play a role cytoskeletal reorganization, cell polarity, morphogenesis, and cell migration (61). These Rho family GTPases are known to affect the turnover of adherens junctions through multiple pathways (148). Both Rho and Rac are required for the establishment of adherens junctions (149, 150), and the activation of Rac has also been shown inhibit desmosomal adhesion in human keratinocytes (151).

The first association of Rho GTPases with PV pathogenesis was seen in experiments which demonstrated that the activation of Rho GTPases could prevent PVIgG-induced blister formation in human skin. Additional studies were able to show that RhoA activation was able to block the PVIgG-induced retraction of KIFs as well as loss of cell adhesion in HaCaT cells (41). Also, it was observed that HaCaT cells treated with PVIgG demonstrated a reduction in RhoA activity. In addition, p38MAPK inhibitors were shown to block the PVIgG-induced reduction of RhoA activity (94). These results suggest that PVIgG-induced activation of p38MAPK may induce blister formation, at least in part, by inhibiting the activity of RhoA. Given that the formation of adherens junctions has been shown to be necessary for proper assembly and disassembly of desmosomes (47, 152, 153), the loss of desmosomal adhesion seen in PV may be secondary to the inhibition of adherens junctions caused by RhoA inhibition.

For a summary of evidence in support of PV-associated signaling pathways, see Table 2.

**Table 2:** Evidence in support of pemphigus vulgaris (PV)-associated signaling pathways.

p38MAPK	Ca/protein kinase C (PKC)	Epidermal growth factor receptor (EGFR)	Rho GTPases
– PVIgG induces significant increases in the phosphorylation of p38MAPK, MAPKAP2, and heat-shock protein (Hsp)27 – Treatment of cells with p38MAPK inhibitors prevents PVIgG-induced acantholysis as well as changes in the actin cytoskeleton and the retraction of KIFs from desmosomal attachments – Inhibition of p38MAPK prevents PVIgG-induced phosphorylation of MAPKAP2, Hsp27, and p38MAPK *in vivo* – p38MAPK and Hsp27 are phosphorylated in the lesional skin of PV patients	– PVIgG leads to a rapid increase in intracellular calcium in keratinocytes – Inhibition of PKC prevents acantholysis both *in vitro* and *in vivo* – Inhibition of phospholipase C prevents PVIgG-induced acantholysis, as well as increases in intracellular calcium and PKC activation	– PVIgG leads to the activation of EGFR in keratinocytes – Anti-Dsg3 autoantibodies can also lead to EGFR activation – Inhibition of EGFR prevents PVIgG-induced skin blistering both *in vivo* and in human skin explants	– Activation of Rho GTPases prevents PVIgG-induced blister formation in human skin – Cells treated with PVIgG demonstrate a reduction in RhoA activity – p38MAPK inhibitors block PVIgG-induced reduction of RhoA activity – RhoA activation blocks PVIgG-induced retraction of KIFs as well as loss of cell adhesion in HaCaT cells

## Integrated Model of PV autoAb-Induced Signaling

The pathogenic processes following the binding of autoAbs that eventually drive the loss of cell adhesion are diverse and complex. Taken together, the above data suggest that the disease mechanisms underlying PV are the result of both the direct steric interference of adhesion molecule interaction by autoAb binding *and* the activation of intracellular signaling pathways elicited *via* autoAb binding. A viewpoint that could tie both these mechanisms together is to see the desmosome/keratin intermediate filament complex as a signaling complex which participates in mechanosensing in addition to providing structural stability. Although desmosomal proteins have not yet been shown to function in this manner, a wealth of data exists which describes a similar function in adherens junctions (154). If this was shown to be the case with desmosomal proteins as well, it would be reasonable to assume that, in addition to physically interfering with desmosomal adhesion, the binding of autoAbs in PV may alter signaling pathways associated with the desmosomal complex, such as p38MAPK, EGFR, and PKC that ultimately interfere with the structural stability of keratinocytes.

**Figure 1:**
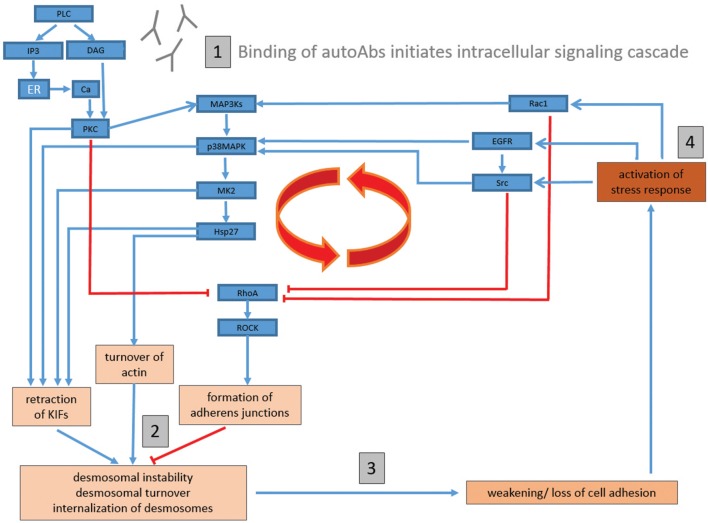
Proposed comprehensive model of signaling mechanisms underlying blister formation in pemphigus vulgaris. (1) Binding of autoantibodies (autoAbs) to target keratinocyte antigens (either desmosomal or extra-desmosomal) induces the activation of phospholipase C (PLC), and subsequently the activation of protein kinase C (PKC) *via* Ca^2+^ and diacylglycerol (DAG), which then activates p38MAPK *via* MAP3ks (such as Ask1); (2) activated PKC {either through direct phosphorylation of keratin filaments or DP} and p38MAPK {*via* MAPKAP2 (MK2) and heat-shock protein (Hsp)27 phosphorylation} then induce the retraction of the KIFs as well as the turnover of the actin cytoskeleton; (3) the retraction of KIFs from the desmosomal plaques, as well as cytoskeletal rearrangements, then cause a destabilization of desmosomes, and a weakening of cell adhesion; (4) finally, the weakening of cell adhesion, coupled with the mechanical stress induced by cytoskeletal rearrangements, induces a cellular stress response, resulting in the activation of Src, epidermal growth factor receptor (EGFR), and Rac1 (as well as other pathways), and re-initiation/perpetuation of the pathological cycle. RhoA is inhibited by multiple pathways (such as PKC, EGFR/Src, and Rac1). As RhoA activation has been shown to inhibit PVIgG-induced acantholysis, an inhibition of RhoA would promote cell dissociation. Blue arrow-headed lines indicate activation, while red bar-headed lines indicate inhibition of selected molecules/pathways.

Taking into consideration the breadth of experimental data detailing autoAb-induced activation of intracellular signaling pathways, we propose that blister formation in PV may result from the following mechanism: (1) binding of autoAbs to target desmosomal antigens (either desmosomal or extra-desmosomal) induces the activation of PLC, leading to the activation of PKC *via* Ca^2+^ and DAG, which in turn activates p38MAPK *via* MAP3ks [such as Ask1 (155, 156)]; (2) activated PKC (either through direct phosphorylation of keratin filaments or DP) and p38MAPK (*via* MK2 and Hsp27 phosphorylation) then induce the retraction of the KIFs as well as the turnover of the actin cytoskeleton; (3) the retraction of KIFs from the desmosomal plaques, as well as cytoskeletal rearrangements, then cause a destabilization of desmosomes, and a weakening of cell adhesion; (4) finally, the weakening of cell adhesion, coupled with the mechanical stress induced by cytoskeletal rearrangements, induces a cellular stress response, resulting in the activation of Src, EGFR, and Rac1 (as well as other pathways), and re-initiation/perpetuation of the pathological cycle (Figure 1).

A primary advantage of this model is the ability to provide a mechanistic framework for understanding how the widely variegated set of factors that have been implicated across multiple studies may be contributing to PV pathogenesis. For example, multiple studies have linked increased levels of reactive oxygen species (ROS) to PV (157–161), but a potential mechanistic contribution to disease has not been well defined. Using our model, however, the known ability of ROS to affect KIF and actin cytoskeletons as well as PKC, Src, p38MAPK, and RhoA signaling (162–167) potentially demonstrates how ROS may contribute to blister formation in a number of ways. As a result, our model can also explain how anti-mitochondrial autoAbs, which have been suggested to play a role in PV and shown to increase ROS production, can directly mediate disease pathogenesis (50, 168–172). Furthermore, the activation of pro-apoptotic pathways by autoAb binding has been shown to modulate p38MAPK (50) and may further contribute to the signaling pathologies that drive acantholysis.

Although this model lays a comprehensive framework for the mechanism of blister formation incorporating all of the major signaling pathways implicated in pathogenesis by the literature, future experiments are required to test the validity of this model and more precisely define the series of signaling events which occur downstream of autoAb binding. Determining the degree to which both desmosomal and extra-desmosomal Dsg proteins associate with signaling molecules and whether or not the binding of autoAbs to these proteins is sufficient to elicit intracellular signaling is a high priority to ascertain if these molecules could function as signal transducers and if so, through which pathways. Immunofluorescent studies of keratinocyte monolayers before and after PVIgG treatment may be one way to determine which molecules are physically associated with desmosomal proteins and determine if autoAb binding leads to their activation/translocation. Another important step would be to more precisely define the upstream MAPK cascade that leads to p38MAPK activation. There are multiple upstream signaling molecules which are known to activate p38MAPK and the characterization of the molecules involved in this process could allow for a better understanding of how autoAbs, or additional factors such as hormones or cytokines (which are known to utilize specific cascades) could activate p38MAPK (173–175). Protein microarray analysis, which allows for rapid, highly specific, multiplexed analysis of the signal transduction pathways (176, 177), would be well suited for this task.

Desmosomal proteins as a mechanosensing signaling complex would provide a logical intersection between the two primary theories of autoAb-induced blister formation in PV. Although future studies are still needed, there exists a great potential to significantly and directly affect the development of future treatments in PV. Identification of the signals transduced by autoAb binding could lead to the identification of potentially novel drug targets, or, at the very least allow researchers to focus on disease-specific signaling pathologies, resulting in small molecule inhibitors with a lower chance of harmful side effects and the ability to minimize systemic suppression of the immune system. In addition, large scale profiling of the signal transduction pathways upstream of MAPK may represent an especially beneficial endeavor as such studies could identify cytokines or hormones which may be contributing to disease process. In addition to facilitating the use of disease specific anti-cytokine or hormonal therapies which may already be in use for other disease modalities, such information could be individualized to the specific pathologies driving disease in a given patient, informing more tailored and effective treatment strategies and greatly enhancing clinical management of disease.

## Author Contributions

AS and TS contributed to the conceptualization and writing of the manuscript.

## Conflict of Interest Statement

The authors declare that the research was conducted in the absence of any commercial or financial relationships that could be construed as a potential conflict of interest.
